# Plasma neurofilament light levels are associated with risk of disability in multiple sclerosis

**DOI:** 10.1212/WNL.0000000000009571

**Published:** 2020-06-09

**Authors:** Ali Manouchehrinia, Pernilla Stridh, Mohsen Khademi, David Leppert, Christian Barro, Zuzanna Michalak, Pascal Benkert, Jan Lycke, Lars Alfredsson, Ludwig Kappos, Fredrik Piehl, Tomas Olsson, Jens Kuhle, Ingrid Kockum

**Affiliations:** From the Department of Clinical Neuroscience (A.M., P.S., M.K., F.P., T.O., I.K.), The Karolinska Neuroimmunology & Multiple Sclerosis Centre, Karolinska Institutet; Centre for Molecular Medicine (A.M., P.S., M.K., F.P., T.O., I.K.), Karolinska University Hospital, Stockholm, Sweden; Departments of Medicine, Biomedicine, and Clinical Research (D.L., C.B., Z.M., L.K., J.K.), Neurologic Clinic and Policlinic, University Hospital Basel, University of Basel; Clinical Trial Unit (P.B.), Department of Clinical Research, University Hospital Basel, University of Switzerland; Institution of Neuroscience and Physiology (J.L.), Sahlgrenska Academy, University of Gothenburg, Gothenburg; Institute of Environmental Medicine (L.A.), Karolinska Institutet, Stockholm; and Centre for Occupational and Environmental Medicine (L.A.), Stockholm County Council, Sweden.

## Abstract

**Objective:**

To investigate the association between plasma neurofilament light chain (pNfL) levels and the risk of developing sustained disability worsening.

**Methods:**

Concentrations of pNfL were determined in 4,385 persons with multiple sclerosis (MS) and 1,026 randomly selected population-based sex- and age-matched controls using the highly sensitive Single Molecule Array (SimoaTM) NF-Light Advantage Kit. We assessed the impact of age-stratified pNfL levels above the 80th, 95th, and 99th percentiles among controls on the risk of Expanded Disability Status Scale (EDSS) worsening within the following year and reaching sustained EDSS scores of 3.0, 4.0, and 6.0 and conversion to secondary progressive multiple sclerosis (SPMS).

**Results:**

The median (interquartile range [IQR]) pNfL was 7.5 (4.1) pg/mL in controls and 11.4 (9.6) pg/mL in MS (*p* < 0.001). The median (IQR) duration of follow-up was 5 (5.1) years. High pNfL was associated with increased adjusted rates of EDSS worsening ranging between 1.4 (95% confidence intervals [CIs]: 1.1–1.8) and 1.7 (95% CI: 1.4–2.3). High pNfL was also associated with the risk of reaching a sustained EDSS score of 3.0, with adjusted rates ranging between 1.5 (95% CI: 1.2–1.8) and 1.55 (95% CI: 1.3–1.8) over all percentile cutoffs (all *p* < 0.001). Similar increases were observed for the risk of sustained EDSS score 4.0. In contrast, the risk of reaching sustained EDSS score 6.0 and conversion to SPMS was not consistently significant.

**Conclusions:**

Elevated pNfL levels at early stages of MS are associated with an increased risk of reaching sustained disability worsening. Hence, pNfL may serve as a prognostic tool to assess the risk of developing permanent disability in MS.

Multiple sclerosis (MS) is an immune-mediated neurodegenerative disease of the CNS, with a highly variable interindividual disease course. As a result, even if using existing clinical prognostic factors, the forecasting of long-term prognosis is often unreliable and imprecise. The increasing number of disease-modifying treatments (DMTs) during the past decades underscores the need for prognostic biomarkers that can aid treatment decisions. This is of particular importance at the earliest phases of the disease, when treatments are most effective, and may affect long-term disability outcomes.^1^

Neurofilaments provide structural support to axons as the main component of the neuronal cytoskeleton. Elevated levels of these proteins have been observed in several neurodegenerative diseases.^2–4^ In persons with MS, elevated CSF levels of neurofilament light chain (NfL) or heavy chain have been associated with brain atrophy and long-term outcome.^5,6^ The less invasively measured serum neurofilament light chain (sNfL) levels have been found to be highly correlated with CSF NfL levels.^7–9^ The need for invasive CSF sampling makes recent progress in developing more sensitive assays that reliably can determine NfL concentrations in blood highly encouraging.^8,10^ However, the potential of sNfL levels to predict long-term outcomes has not been well studied. The objective of this study was to explore the association between plasma neurofilament light chain (pNfL) sampled early in the disease course and long-term clinical disability outcomes using a large population-based MS sample with matched controls.

## Methods

### Study population

Our study population included a subset of MS cases and controls participating in 2 prospective large cohorts, the Epidemiological Investigation of Multiple Sclerosis (EIMS)^11^ and Immunomodulation and Multiple Sclerosis Epidemiology (IMSE) cohorts.^12^ In the EIMS study, individuals with newly diagnosed MS aged between 16 and 70 years are identified at neurology clinics throughout Sweden and invited to participate in the study by completing a questionnaire and donating a blood sample. All cases have been examined by a neurologist at the clinic where they were recruited, and only patients fulfilling the McDonald criteria^13–15^ are included in the study cohort. For each patient in the EIMS cohort, at least 2 age (5-year intervals)-, sex-, and residential area (county)-matched randomly selected controls are identified by Statistics Sweden and are invited to participate in the study by completing a questionnaire and donating a blood sample. If neither of the selected controls responds to the questionnaire, other matched controls are contacted. The IMSE cohorts are part of a nationwide phase 4 surveillance study aimed at investigating the long-term safety and efficacy of all more recent DMTs starting from natalizumab. Combined, these cohorts contribute additional data beyond what are collected in the Swedish MS register (SMSreg), including DNA and plasma samples.

The SMSreg provides the platform for longitudinal clinical follow-up of MS cases participating in both cohorts.^12,16^ Data collected include date of MS onset and diagnosis, disease course (relapsing-remitting or primary/secondary progressive [SP]), disability scores, relapses, DMTs, and laboratory results.

For this study, pNfL was measured in a number of controls (to define the age-standardized percentiles of pNfL) and MS cases. MS cases were required to have been diagnosed with clinically definite MS,^13–15,17^ being included in the SMSreg, and have a biological sample before possible inclusion. Controls should have been genotyped, have a matched case with available genotype data, and have a biological sample to be included. By virtue of design, cases can move between and contribute to the data in different cohorts (e.g., as a result of change in treatment); however, only unique cases were included in this study.

### Sampling and pNfL measurement

In the EIMS cohort, ethylenediaminetetraacetic acid (EDTA)-treated plasma samples were collected at the time of MS diagnosis and at the same age for the corresponding matched controls. In the IMSE cohorts, EDTA plasma samples were collected at baseline before initiation of the DMTs, where patients were either treatment naive or were exposed to only interferons and/or glatiramer acetate. All blood samples were posted by surface mail before separation of plasma.^16^ Plasma samples were stored at −80°C following standard procedures. Concentrations were determined using a sensitive immunoassay on the Simoa platform at the University Hospital Basel using the commercially available NF-Light kit and using antibodies from UmanDiagnostics according to the manufacturer's instructions (Quanterix, Lexington, MA). Measurements and storage were performed on coded samples in the same manner for cases and controls. The samples were run in 2 batches were EIMS and IMSE cases were split between the 2. EIMS cases and their matched controls were run in the first batch together. The laboratory personnel had no access to clinical data and remained blinded to treatment allocation and diagnosis.

### Study outcomes

In the SMSreg, data are recorded by neurologists or MS nurses through a web interface and include patient characteristics, MS course, DMT exposure, visits, clinical scales (including the Expanded Disability Status Scale [EDSS] score), relapses, MRI, and laboratory tests. Most data are collected at routine clinical visits on annual or biannual basis, but relapses and MRI are recorded at the time of event.^18^

### Early clinical activities and baseline disease severity

Annualized relapse rates (ARRs) prior sampling, nature of the first relapse (sensory vs motor vs combination), and degree of recovery from the first relapse (full vs partial/no recovery after 6 months) were investigated. These analyses were limited to the EIMS cohort because the shorter gap between MS onset and sampling date allowed for a better evaluation of the association between early clinical activities and pNfL levels. We further assessed the association between baseline disease severity as measured by the global Age-Related MS Severity (ARMSS) score^19^ and pNfL levels in all cases with available baseline EDSS scores.

### Risk of sustained disability worsening

The EDSS score is the most common method of quantifying disability in MS. Scoring is performed by a neurologist measuring the impact of MS on 8 functional systems.^20^ In the SMSreg, EDSS examination is performed and recorded by an MS specialist neurologist at annual or biannual clinic visits. The EDSS score milestones 3.0 (moderate disability), 4.0 (significant disability but able to walk without aid or rest for 500 m), and 6.0 (requirement of unilateral assistance to walk about 100 m with or without resting) were the end points in this study. Sustained progression in EDSS milestones was defined as, when met the end point, not reverted to lower EDSS levels until the end of follow-up and having at least 1 subsequent EDSS score being equal to or greater than the end point. Patients not fulfilling this definition were censored at the time of their last EDSS measurement. Those who met the end point at the last observation, but lacked a subsequent disability evaluation after meeting the end point, were censored at the second to last EDSS measurement. This was performed to ensure exclusion of individuals with transient EDSS worsening. Those who met the end point already at baseline were excluded from the analyses (i.e., EDSS scores ≥3.0 or ≥4.0 or ≥6.0 at the first clinic visit).

### Risk of conversion to secondary progressive multiple sclerosis

Conversion to an SP disease course is one of the most significant clinical milestones for long-term disease burden in MS.^21^ In the SMSreg, the year of conversion to secondary progressive multiple sclerosis (SPMS) in relapsing-onset patients is determined and recorded by the practicing neurologist following the 1996 MS disease course consensus.^22^ Risk of conversion to SP course from the date of sampling was determined in relapsing-onset MS using survival analysis. Patients were followed from the date of sampling to the date of conversion to SPMS or their last clinical visit if they remained RR by the end of follow-up.

### Statistical analysis

We used the Mann-Whitney test for comparison of pNfL levels between groups with regard to the baseline characteristics, baseline ARMSS score, and prebaseline annualized relapse rate. Associations between pNfL levels and age at the time of sampling in controls and MS subtypes (relapsing, secondary, and primary progressive) were modeled using generalized linear regression models on log-transformed pNfL.

We categorized persons with MS according to the age-stratified pNfL levels above and below the 80th (C80), 95th (C95), and 99th (C99) percentiles among controls. The age-specific levels of pNfL in controls were calculated by modeling the distribution using Generalized Additive Models for Location, Scale, and Shape (GAMLSS) with Box-Cox t distribution.^7,23,24^ In MS cases, the association between pNfL levels above vs below the calculated percentiles (>C80, >C95, and >C99) and the short- and long-term outcomes were assessed.

When analyzing the risk of reaching EDSS milestones or SPMS, we calculated the risk of reaching the outcomes from the date of sampling using Cox proportional hazard regression models while adjusting for potential confounders. Confounders included sex, age, and disease duration at the time of sampling, exposure to first-line DMTs (interferon beta-1a, interferon beta-1b, peginterferon beta-1a, glatiramer acetate, teriflunomide, and dimethyl fumarate), exposure to second-line DMTs (natalizumab, fingolimod, rituximab, ocrelizumab, alemtuzumab, and daclizumab), and ARR before the sampling date. Treatments were modeled as time-varying covariates. We also performed a sensitivity analysis of time to disability milestones by running the analysis on patients in the EIMS cohort who had the longest duration of follow-up and were treatment naive at the time of sampling. Two additional sensitivity analyses were performed. In the first sensitivity analysis, we adjusted the models for the baseline EDSS score to investigate the association between increased pNfL and sustained disability worsening in the presence of the baseline EDSS score, which is a strong predictor factor of disability worsening. In the second sensitivity analysis, we adjusted the models for the body mass index (BMI) as we have recently shown that the pNfL is correlated with BMI.^25^ BMI was calculated based on the self-reported weight and height at the time of sampling.

In addition, we investigated the odds of worsening of the EDSS score within the next year following sampling using logistic regression models adjusted for sex, baseline EDSS score, disease duration, disease course (relapsing vs progressive), and age at the time of sampling.

To elaborate on the clinical application of pNfL, we performed the recursive partitioning (rPART) method separately for relapsing and progressive patients.^26^ Briefly, rPART identifies the optimal split of pNfL that would partition the data into 2 outcome groups: “Worsened” (people who experience EDSS worsening within the next year following sampling) and “Stable” (people whose EDSS score remained stable). To generate a simpler tree, pruning based on the complexity parameter was used. Complexity parameter is the minimum improvement in the model needed in each node. We then calculated the sensitivity, specificity, positive predictive value (PPV), and negative predictive value (NPV) of the trees for predicting EDSS worsening within the next year following sampling. R version 3.3.4^27^ and the packages “survival” and “rpart” were used for data management and data analysis.

### Standard protocol approvals, registrations, and patient consents

This study was approved by the Stockholm regional ethical committee (EPN), and all participants had given written informed consent.

### Data availability

Data related to the current article are available from Ingrid Kockum, Karolinska Institutet. To be able to share data from the EIMS and IMSE cohorts, a data transfer agreement needs to be completed between Karolinska Institutet and the institution requesting data access. This is in accordance with the data protection legislation in Europe (General Data Protection Regulation [GDPR]). Persons interested in obtaining access to the data should contact Ali Manouchehrinia (ali.manouchehrinia@ki.se).

## Results

In the EIMS cohort, the response rate to the questionnaire was 89% (95% contributed blood sample) in cases and 60% in controls (37% contributed blood sample). From the 1,714 individuals in the IMSE natalizumab cohort, we included 370 unique patients (not included in any other cohort) with a baseline blood sample. From the total of 525,442 and 602 patients in the dimethyl fumarate, fingolimod and rituximab cohorts 378, 316 and 47 unique patients with baseline and 12-month samples were included. All the 133 and 49 unique patients in the teriflunomide and alemtuzumab cohorts were included.

Overall, a total of 1,026 population-based non-MS controls who were sex and age matched to the participants of the EIMS cohort were included. The corresponding combined MS sample comprised 4,385 persons with MS selected among those with available demographic and clinical data. Interassay coefficients of variation (CVs) for 3 plasma samples were below 13%. The mean intra-assay CV of duplicate determinations for concentration was 6.4%.

### pNfL level in controls and percentile curves

**Figure 1:**
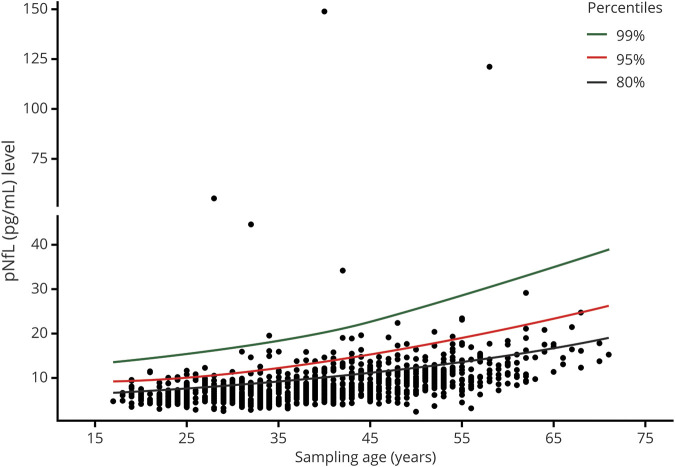
Association between pNfL level and age in population-based controls pNfL percentiles at different ages in 1,026 controls obtained from Generalized Additive Models for Location, Scale, and Shape (GAMLSS) with Box-Cox t distribution. pNfL = plasma neurofilament light chain

A summary of the demographic and clinical data of the study population is presented in table 1. There was a significant association between the pNfL levels and age at the time of sampling where the pNfL level increased by 0.18 pg/mL (95% confidence intervals [CIs]: 0.17–0.20, *p* < 0.001) per year of age. The difference between sexes was not significant (female: 7.50 vs male: 7.85, *p* = 0.2). The calculated pNfL percentiles in controls obtained from GAMLSS are shown in figure 1 and table e-1 (available on Dryad: doi.org/10.5061/dryad.msbcc2ftm).

**Table 1:**
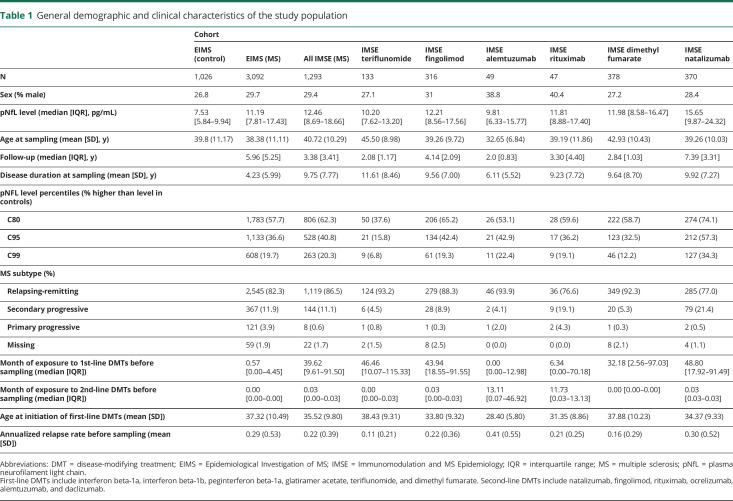
General demographic and clinical characteristics of the study population

### pNfL level in persons with MS

**Figure 2:**
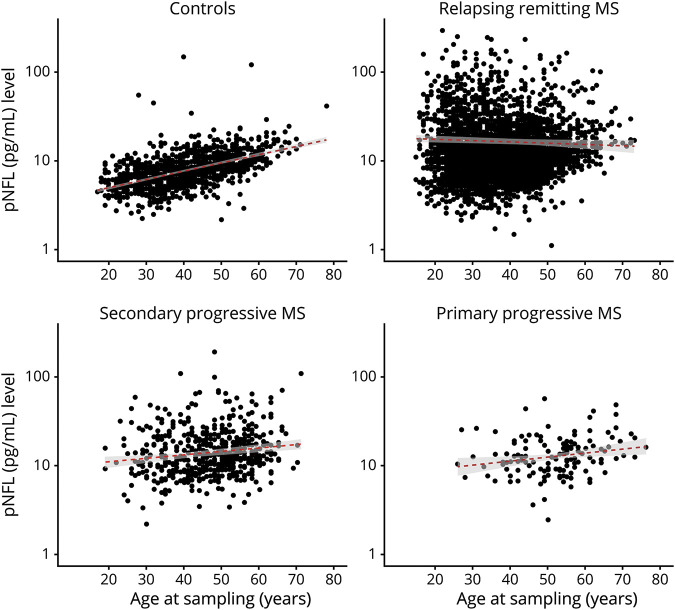
Association between pNfL level and age at the time of sampling in MS cases pNfL levels increased by 0.18 pg/mL (95% CI: 0.17–0.20, *p* < 0.001) per each year of age in controls, by −0.05 pg/mL (95% CI: −0.07 to −0.04, *p* = 0.001) in relapsing remitting patients, by 0.12 pg/mL (95% CI: 0.09–0.16, *p* < 0.001) in SP patients, and by 0.14 pg/mL (95% CI: 0.08–0.19, *p* < 0.001) in primary progressive patients. CI = confidence interval; pNfL = plasma neurofilament light chain; SP = secondary progressive.

**Figure 3:**
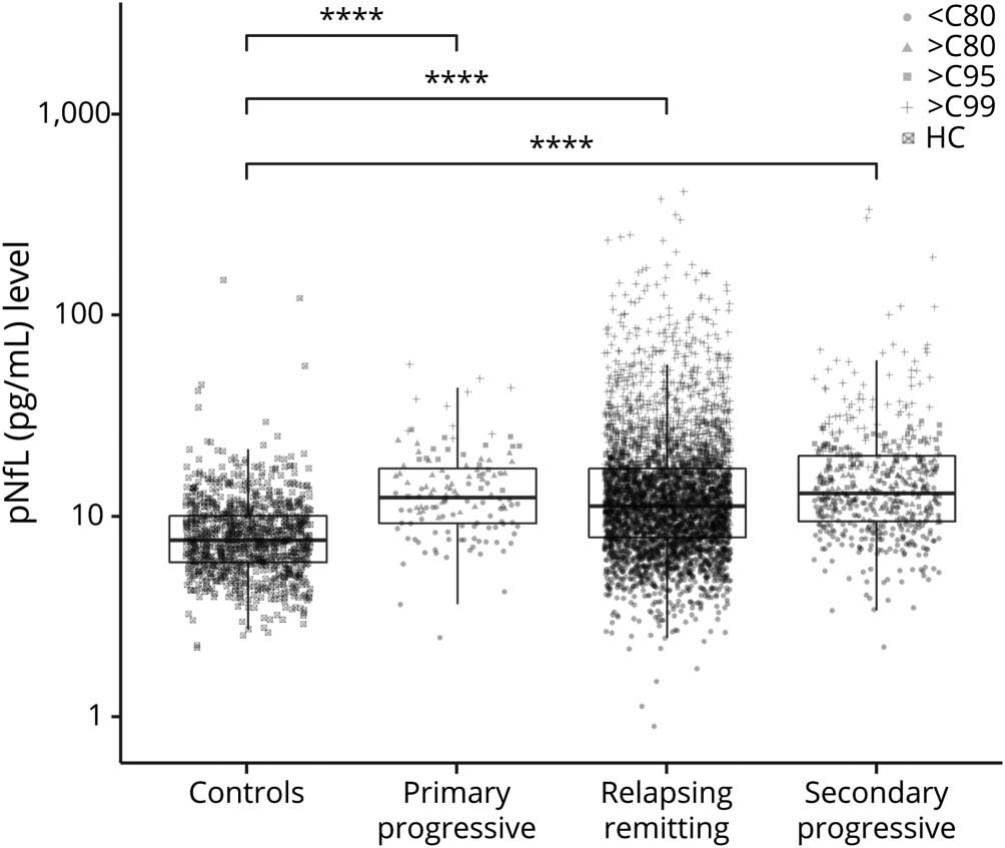
Plasma NfL level comparisons between MS subtypes and population-based controls Comparison of pNfL levels between 1,026 healthy controls, 3,664 patients with relapsing-remitting MS, 511 patients with secondary progressive multiple sclerosis, and 129 patients with primary progressive MS. Significance code: ****<0.0001. MS = multiple sclerosis; pNfL = plasma neurofilament light chain.

In univariate analyses, the pNfL level decreased by −0.05 pg/mL (95% CI: −0.07 to −0.04, *p* = 0.001) in relapsing remitting MS and increased by 0.12 pg/mL (95% CI: 0.09–0.16, *p* < 0.001) in SPMS and by 0.14 pg/mL (95% CI: 0.08–0.19, *p* < 0.001) in PP cases per each year of age (figure 2). The pNfL level was significantly higher in all 3 MS subtypes compared with controls (figure 3). Corrected for the age at the time of sampling, the median pNfL level was 8.50 pg/mL (interquartile range [IQR]: 0.77 pg/mL) in controls, 17.10 pg/mL (IQR: 0.68 pg/mL) in relapsing remitting MS, 18.40 pg/mL (IQR: 0.70 pg/mL) in SPMS, and 14.7 pg/mL (IQR: 0.59 pg/mL) in primary progressive MS.

### Early clinical activity, baseline severity, and pNfL level

The annualized relapse rate in the years before sampling was significantly higher in patients with pNfL levels above vs below the respective calculated percentiles of controls. There was no clinically meaningful association between degree of recovery from the first relapse, nature of the first relapse (sensory vs motor vs combination), and pNfL levels. Disease severity, as measured by the global ARMSS score at baseline, was significantly associated with the pNfL level. The median unadjusted global ARMSS score increased significantly with the increase in the pNfL levels (table 2).

**Table 2:**
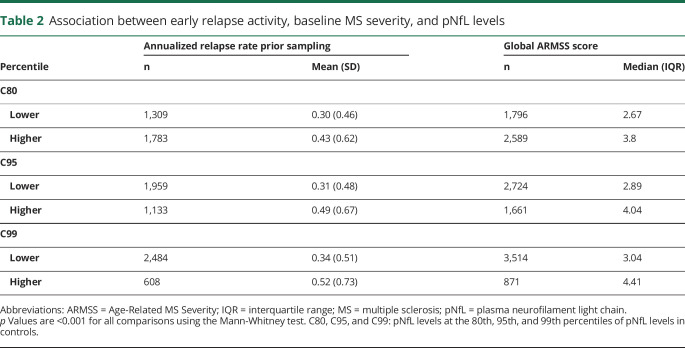
Association between early relapse activity, baseline MS severity, and pNfL levels

### Risk of sustained EDSS worsening

The median duration of follow-up was 5 years (IQR: 5.1 years). A total of 3,370 patients were included in the analysis of risk of reaching sustained EDSS score 3.0, and 525 (15.5%) reached this outcome. A total of 3,840 and 3,990 patients were included in the analyses of risk of reaching sustained EDSS scores 4.0 and 6.0, of whom 352 (9.1%) and 199 (5.0%) had reached these milestones, respectively.

**Figure 4:**
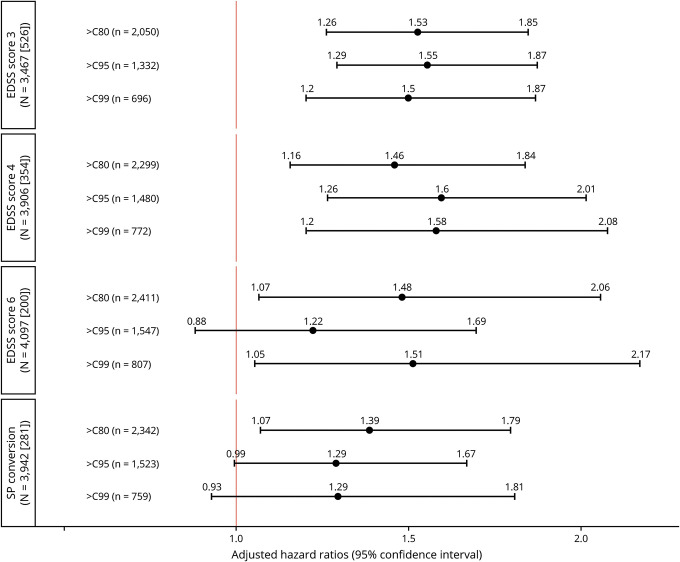
Risk of reaching major disability milestones stratified by baseline pNfL levels Adjusted hazard ratios (95% confidence intervals) and *p* value for risks of conversion to secondary progressive multiple sclerosis (SPMS) type, reaching sustained EDSS scores 3.0, 4.0, and 6.0 in patients at different pNfL levels. >C80, >C95, and >C99: pNfL levels higher than the 80th, 95th, and 99th percentiles of pNfL levels in controls. Numbers in the box denote the number of patients included in each analysis (number of events). EDSS = Expanded Disability Status Scale; pNfL = plasma neurofilament light chain.

We found no significant impact of the ARR before the sampling date on the risk of reaching EDSS milestones (data not shown). Hence, the final models were controlled for sex, age, and disease duration at the time of sampling and exposure to first- and second-line DMTs (defined in the Methods section). The adjusted hazard ratio (HR) of reaching a sustained EDSS score of 3.0 was 1.49 (95% CI: 1.23–1.81, *p* < 0.001) for pNfL level >C80. The HRs were 1.51 (95% CI: 1.25–1.82, *p* < 0.001) and 1.47 (95% CI: 1.18–1.83, *p* < 0.001) for pNfL levels >C95 and >C99, respectively. A similar increase was observed in the risk of reaching a sustained EDSS score of 4.0. The risk of reaching a sustained EDSS score of 6.0 was significantly increased in the groups with pNfL levels >C80 and >C99 (figure 4). The results remained essentially the same across the 3 EDSS outcomes when analyses were limited to patients in the EIMS cohort only (data not shown), when the models were adjusted for the baseline EDSS score or baseline BMI (figure e-1 available on Dryad: doi.org/10.5061/dryad.msbcc2ftm).

Furthermore, we found an increased risk of EDSS worsening in the year after sampling in all pNfL percentiles. The adjusted ratios were 1.40 (95% CI: 1.10–1.78), 1.65 (95% CI: 1.30–2.09), and 1.52 (95% CI: 1.16–1.99) for >C80, >C95, and >C99, respectively. Similar results were found when analysis was limited to the progressive group (data not shown).

### Risk of conversion to SPMS

From the 3,824 relapsing-onset patients, 279 (7.3%) had converted to SPMS during the follow-up time. Overall, there was a consistent but marginal increase in the risk of transitioning to SPMS among cases with elevated baseline pNfL levels. The adjusted HRs for transitioning to SPMS were 1.39 (95% CI: 1.07–1.79, *p* = 0.01), 1.29 (95% CI: 0.99–1.67, *p* = 0.055), and 1.29 (95% CI: 0.92–1.80, *p* = 0.13) for pNfL levels >C80, >C95, and >C99, respectively (figure 4). No significant increase in the risk of conversion to SPMS was seen when models were adjusted for the baseline EDSS score or BMI (figure e-1 available on Dryad: doi.org/10.5061/dryad.msbcc2ftm).

### Predictive value and the optimal cutoffs of pNfL

We included 1,932 MS cases, 1,638 relapsing-remitting and 294 SP, with available data. The optimal cutoff values were 15 pg/mL in relapsing-remitting MS and 8.5 pg/mL in SP cases. Among relapsing-remitting patients with pNfL level <15 pg/mL (69% of all relapsing cases), the class prediction was “Stable” with the probability of next year worsening of 17%. The class prediction was “Worsening” in 3% of the cases with pNfL ≥15 pg/mL. Among SP cases with pNfL level <8.5 pg/mL (20% of SP cases), the class prediction was “Stable” with worsening probability of 14%. The class prediction was “Worsening” in 10% of the cases with pNfL level ≥8.5 pg/mL (figure e-2 available on Dryad: doi.org/10.5061/dryad.msbcc2ftm). The accuracy of decision tree was 0.81 (95% CI: 0.79–0.83) in relapsing MS and 0.76 (95% CI: 0.71–0.81) in SP type. The sensitivity, specificity, PPV, and NPV were 0.99, 0.08, 0.82, and 0.61 in relapsing and 0.95, 0.23, 0.77, and 0.64 in SP type, respectively.

## Discussion

Using large nationwide population-based data, we find that elevated pNfL levels at the time of MS diagnosis are associated with the risk of long-term sustained disability development. MS patients with pNfL concentrations >80 percentile levels in persons without MS were at higher risk of reaching sustained EDSS milestones 3.0, 4.0, and, 6.0. In some of the analyzed strata, elevated levels of pNfL were also associated with a higher risk of transitioning to the progressive MS phase in relapsing-onset patients. However, this was not consistently significant. Notably, these associations remained significant after controlling for major confounders, including baseline EDSS score, BMI, exposure to DMTs, and age. These findings suggest that pNfL measurement can usefully provide additional predictive power in the form of an easily accessible and easy-to-measure biomarker for monitoring of disease activity and potentially treatment response in MS.

Existing data on the relation between NfL and clinical outcomes in MS are limited to illustrations of relatively short-term correlations.^7,9,24,28–34^ Our study validates and extends these findings by examining the long-term association between elevated pNfL levels and disability worsening in a large nationwide population-based cohort of patients with MS with diverse clinical characteristics. In addition, we described NfL levels in 1,026 population-based control group, further defining the age-related increase of pNfL. This is most important when considering the values obtained from persons with MS at different ages.

Great emphasis has recently been placed on the need for predictive tools to assess future disease worsening in MS. Currently, an obvious issue in developing decision aid tools in MS is a lack of accurate, standardized, and readily measured biomarkers of disease worsening and progression.^35^ Our findings suggest that pNfL is useful as a prognostic biomarker for predicting important disability outcomes and sustained disability in MS. Added benefits of a blood biomarker such as NfL over imaging biomarkers such as brain volumetric measurements or clinical markers such as baseline severity levels are its longitudinal and repeated accessibility, lower costs, and greater potential for standardization. Neurofilament light chain in blood is likely to reflect an integral measure of recent and ongoing neuronal damage. Moreover, NfL comprises spinal cord pathology in addition to the brain and offers a real-time measure as opposed to MRI, which is retrospective.

Overall, pNfL showed high sensitivity and low specificity for identifying individuals at risk of short-term disability worsening, indicating that individuals with pNfL levels above the cutoffs are more likely to experience EDSS worsening within the next year after sampling. The demonstrated predictive relevance of pNfL on the group level is an important prerequisite for further developments of pNfL toward a treatment monitoring tool in individual patients and other personalized medicine applications.

The strengths of this study include use of data from a large population-based cohort of patients with MS, longitudinal follow-up, and, importantly, a large and population-based group of controls. Nonetheless, our study has limitations. We observed a significant variability and overlap in the pNfL levels between controls and persons with MS and within different MS phenotypes. It is likely that pNfL levels are also influenced by other factors, e.g., comorbidities, that we could not analyze here. Also, we did not have access to sufficiently precise long-term MRI data to be included here. Additional studies are therefore needed to refine the impact of potential confounding factors to improve the MS-specific prognostic power of variability in NfL levels. Moreover, despite the large number of patients included in each analysis, the overall number of patients who reached the sustained clinical milestones was small. This somewhat affected our risk estimates, specifically in the analyses of time to an EDSS score of 6.0 and conversion to SPMS.

In conclusion, elevated pNfL levels measured early on in the disease course are associated with an increased risk of reaching sustained disability milestones. Hence, pNfL may serve as a prognostic and treatment monitoring tool to assess the risk of developing permanent disability in MS as part of a more standardized, noninvasive, longitudinally accessible, and generalizable approach.

## Disclosures

A. Manouchehrinia and P. Stridh were supported by the Margaretha af Ugglas Foundation. M. Khademi and D. Leppert report no disclosures. C. Barro received conference travel grant from Novartis. Z. Michalak and P. Benkert report no disclosures. J. Lycke has received travel support and/or lecture honoraria from Biogen, Novartis, Teva, and Genzyme/Sanofi-Aventis; has served on scientific advisory boards for Almirall, Teva, Biogen, Novartis, and Genzyme/Sanofi-Aventis; serves on the editorial board of *Acta Neurologica Scandinavica*; and has received unconditional research grants from Biogen, Novartis, and Teva. L. Alfredsson has received lecture honoraria from Biogen and Teva. L. Kappos' Institution (University Hospital Basel) received in the last 3 years and used exclusively for research support at the Department: steering committee, advisory board, and consultancy fees from Actelion, Alkermes, Almirall, Bayer, Biogen, Celgene/Receptos, df-mp, Excemed, GeNeuro SA, Genzyme, Japan Tobacco, Merck, Minoryx, Mitsubishi Pharma, Novartis, Roche, Sanofi-Aventis, Santhera, Teva, and Vianex and royalties for Neurostatus-UHB products. The Research of the MS Center in Basel has been supported by grants from Bayer, Biogen, Novartis, the Swiss MS Society, the Swiss National Research Foundation, the European Union, and Roche Research Foundations. F. Piehl has received research grants from Biogen, Genzyme, Merck KGaA, and Novartis and fees for serving as Chair of DMC in clinical trials with Parexel. T. Olsson has received unrestricted MS research grants and/or lecture advisory board honoraria from Biogen, Novartis, Sanofi, and Roche. J. Kuhle served on scientific advisory boards for Novartis Pharmaceuticals, Merck, Biogen, Sanofi Genzyme, Roche, and Bayer; has received funding for travel and/or speaker honoraria from Biogen, Sanofi Genzyme, Novartis, Merck Serono, Roche, Teva, and the Swiss MS Society; and has received research support from Bayer, Biogen, Merck, Sanofi Genzyme, Novartis, Roche, ECTRIMS Research Fellowship Programme, University of Basel, the Swiss MS Society, and the Swiss National Research Foundation (320030_189140/1). I. Kockum is supported by Horizon 2020 MultipleMS grant no. 733161. Go to Neurology.org/N for full disclosures.

## Glossary

ARMSS: Age-Related MS Severity
ARR: annualized relapse rate
BMI: body mass index
CI: confidence interval
CV: coefficient of variation
DMT: disease-modifying treatment
EDSS: Expanded Disability Status Scale
EDTA: ethylenediaminetetraacetic acid
GAMLSS: Generalized Additive Models for Location, Scale, and Shape
HR: hazard ratio
EIMS: Epidemiologic Investigation of Multiple Sclerosis
IMSE: Immunomodulation and Multiple Sclerosis Epidemiology
IQR: interquartile range
MS: multiple sclerosis
NfL: neurofilament light chain
NPV: negative predictive value
pNfL: plasma neurofilament light chain
PPV: predictive positive value
rPART: recursive partitioning
sNfL: serum neurofilament light chain
SP: secondary progressive

## References

[1] Kavaliunas A, Manouchehrinia A, Stawiarz L, et al. Importance of early treatment initiation in the clinical course of multiple sclerosis. Mult Scler 2017;23:1233–1240. 27754943 10.1177/1352458516675039

[2] Mattsson N, Andreasson U, Zetterberg H, Blennow K; Initiative for the ADN. Association of plasma neurofilament light with neurodegeneration in patients with Alzheimer disease. JAMA Neurol 2017;74:557–566. 28346578 10.1001/jamaneurol.2016.6117 PMC5822204

[3] Bacioglu M, Maia LF, Preische O, et al. Neurofilament light chain in blood and CSF as marker of disease progression in mouse models and in neurodegenerative diseases. Neuron 2016;91:56–66. 27292537 10.1016/j.neuron.2016.05.018

[4] Khalil M, Teunissen CE, Otto M, et al. Neurofilaments as biomarkers in neurological disorders. Nat Rev Neurol 2018;14:577–589. 30171200 10.1038/s41582-018-0058-z

[5] Petzold A, Steenwijk MD, Eikelenboom JM, Wattjes MP, Uitdehaag BM. Elevated CSF neurofilament proteins predict brain atrophy: a 15-year follow-up study. Mult Scler J 2016;22:1154–1162. 10.1177/1352458516645206 27207456

[6] Salzer J, Svenningsson A, Sundstrom P. Neurofilament light as a prognostic marker in multiple sclerosis. Mult Scler 2010;16:287–292. 20086018 10.1177/1352458509359725

[7] Disanto G, Barro C, Benkert P, et al. Serum Neurofilament light: a biomarker of neuronal damage in multiple sclerosis. Ann Neurol 2017;81:857–870. 28512753 10.1002/ana.24954 PMC5519945

[8] Piehl F, Kockum I, Khademi M, et al. Plasma neurofilament light chain levels in patients with MS switching from injectable therapies to fingolimod. Mult Scler J 2018;24:1046–1054. 10.1177/1352458517715132 28627962

[9] Novakova L, Axelsson M, Khademi M, et al. Cerebrospinal fluid biomarkers as a measure of disease activity and treatment efficacy in relapsing-remitting multiple sclerosis. J Neurochem 2017;141:296–304. 27787906 10.1111/jnc.13881

[10] Kuhle J, Barro C, Andreasson U, et al. Comparison of three analytical platforms for quantification of the neurofilament light chain in blood samples: ELISA, electrochemiluminescence immunoassay and Simoa. Clin Chem Lab Med 2016;54:1655–1661. 27071153 10.1515/cclm-2015-1195

[11] Hedström AK, Hillert J, Olsson T, Alfredsson L. Smoking and multiple sclerosis susceptibility. Eur J Epidemiol 2013;28:867–874. 24146047 10.1007/s10654-013-9853-4 PMC3898140

[12] Holmén C, Piehl F, Hillert J, et al. A Swedish national post-marketing surveillance study of natalizumab treatment in multiple sclerosis. Mult Scler J 2011;17:708–719. 10.1177/1352458510394701 21228027

[13] McDonald WI, Compston A, Edan G, et al. Recommended diagnostic criteria for multiple sclerosis: guidelines from the International Panel on the Diagnosis of Multiple Sclerosis. Ann Neurol 2001;50:121–127. 11456302 10.1002/ana.1032

[14] Polman CH, Reingold SC, Edan G, et al. Diagnostic criteria for multiple sclerosis: 2005 revisions to the “McDonald Criteria.” Ann Neurol 2005;58:840–846. 16283615 10.1002/ana.20703

[15] Polman CH, Reingold SC, Banwell B, et al. Diagnostic criteria for multiple sclerosis: 2010 revisions to the McDonald criteria. Ann Neurol 2011;69:292–302. 21387374 10.1002/ana.22366 PMC3084507

[16] Frisell T, Forsberg L, Nordin N, et al. Comparative analysis of first-year fingolimod and natalizumab drug discontinuation among Swedish patients with multiple sclerosis. Mult Scler 2016;22:85–93. 25921036 10.1177/1352458515579216

[17] Thompson AJ, Banwell BL, Barkhof F, et al. Diagnosis of multiple sclerosis: 2017 revisions of the McDonald criteria. Lancet Neurol 2018;17:162–173. 29275977 10.1016/S1474-4422(17)30470-2

[18] Alping P, Piehl F, Langer-Gould A, Frisell T; COMBAT-MS Study Group. Validation of the Swedish Multiple Sclerosis Register: further improving a resource for pharmacoepidemiologic evaluations. Epidemiology 2019;30:230–233. 30721167 10.1097/EDE.0000000000000948 PMC6369893

[19] Manouchehrinia A, Westerlind H, Kingwell E, et al. Age related multiple sclerosis severity score: disability ranked by age. Mult Scler J 2017;23:1938–1946. 10.1177/1352458517690618 PMC5700773 28155580

[20] Kurtzke JF. Rating neurologic impairment in multiple sclerosis: an expanded disability status scale (EDSS). Neurology 1983;33:1444–1452. 6685237 10.1212/wnl.33.11.1444

[21] Rovaris M, Confavreux C, Furlan R, Kappos L, Comi G, Filippi M. Secondary progressive multiple sclerosis: current knowledge and future challenges. Lancet Neurol 2006;5:343–354. 16545751 10.1016/S1474-4422(06)70410-0

[22] Lublin FD, Reingold SC. Defining the clinical course of multiple sclerosis: results of an International Survey. National Multiple Sclerosis Society (USA) Advisory Committee on Clinical Trials of new agents in multiple sclerosis. Neurology 1996;46:907–911. 8780061 10.1212/wnl.46.4.907

[23] Rigby RA, Stasinopoulos DM. Generalized additive models for location, scale and shape (with discussion). J R Stat Soc Ser C 2005;54:507–554.

[24] Barro C, Benkert P, Disanto G, et al. Serum neurofilament as a predictor of disease worsening and brain and spinal cord atrophy in multiple sclerosis. Brain 2018;141:2382–2391. 29860296 10.1093/brain/awy154

[25] Manouchehrinia A, Piehl F, Hillert J. Confounding effect of blood volume and body mass index on blood neurofilament light chain levels. Ann Clin Transl Neurol 2020:139–143. 31893563 10.1002/acn3.50972 PMC6952306

[26] Breiman L, Friedman JH, Jerome H, Olshen RA, Stone CJ. Classification and Regression Trees. Chapman & Hall/CRC: Boca Raton, FL; 1984.

[27] R Core Team. R: A Language and Environment for Statistical Computing. Version 3.3.2. Vienna, Austria: R Found Stat Comput; 2016.

[28] Kuhle J, Barro C, Disanto G, et al. Serum neurofilament light chain in early relapsing remitting is increased and correlates with CSF levels and with MRI measures of disease severity. Mult Scler 2016;22:1550–1559. 26754800 10.1177/1352458515623365

[29] Siller N, Kuhle J, Muthuraman M, et al. Serum neurofilament light chain is a biomarker of acute and chronic neuronal damage in early multiple sclerosis. Mult Scler J 2018;25:678–686. 10.1177/1352458518765666 29542376

[30] Quintana E, Coll C, Salavedra-Pont J, et al. Cognitive impairment in early stages of multiple sclerosis is associated with high cerebrospinal fluid levels of chitinase 3-like 1 and neurofilament light chain. Eur J Neurol 2018;25:1189–1191. 29797629 10.1111/ene.13687

[31] Lycke JN, Karlsson JE, Andersen O, Rosengren LE. Neurofilament protein in cerebrospinal fluid: a potential marker of activity in multiple sclerosis. J Neurol Neurosurg Psychiatry 1998;64:402–404. 9527161 10.1136/jnnp.64.3.402 PMC2170011

[32] Novakova L, Zetterberg H, Sundström P, et al. Monitoring disease activity in multiple sclerosis using serum neurofilament light protein. Neurology 2017;89:2230–2237. 29079686 10.1212/WNL.0000000000004683 PMC5705244

[33] Malmeström C, Haghighi S, Rosengren L, Andersen O, Lycke J. Neurofilament light protein and glial fibrillary acidic protein as biological markers in MS. Neurology 2003;61:1720–1725. 14694036 10.1212/01.wnl.0000098880.19793.b6

[34] Novakova L, Axelsson M, Khademi M, et al. Cerebrospinal fluid biomarkers of inflammation and degeneration as measures of fingolimod efficacy in multiple sclerosis. Mult Scler 2017;23:62–71. 27003946 10.1177/1352458516639384

[35] Manouchehrinia A, Zhu F, Piani-Meier D, et al. Predicting risk of secondary progression in multiple sclerosis: a nomogram. Mult Scler J 2018;25:1102–1112. 10.1177/1352458518783667 29911467

